# Changes in Remotely Sensed Vegetation Growth Trend in the Heihe Basin of Arid Northwestern China

**DOI:** 10.1371/journal.pone.0135376

**Published:** 2015-08-18

**Authors:** Wenchao Sun, Hao Song, Xiaolei Yao, Hiroshi Ishidaira, Zongxue Xu

**Affiliations:** 1 College of Water Sciences, Beijing Normal University, Beijing, China; 2 Joint Center for Global Change Studies (JCGCS), Beijing, China; 3 Interdisciplinary Graduate School of Medicine and Engineering, University of Yamanashi, Kofu, Yamanashi, Japan; Chinese Academy of Forestry, CHINA

## Abstract

The Heihe River Basin (HRB) is the second largest inland river basin in China, characterized by high diversity in geomorphology and irrigated agriculture in middle reaches. To improve the knowledge about the relationship between biotic and hydrological processes, this study used Global Inventory Modeling and Mapping Studies Normalized Difference Vegetation Index (NDVI) data (1982–2006) to analyze spatiotemporal variations in vegetation growth by using the Mann—Kendall test together with Sen’s slope estimator. The results indicate that 10.1% and 1.6% of basin area exhibit statistically significant (p < 0.05) upward and downward trends, and maximum magnitude is 0.066/10a and 0.026/10a, respectively. More specifically, an increasing trend was observed in the Qilian Mountains and Hexi Corridor and a decreasing trend detected in the transitional region between them. Increases in precipitation and temperature may be one possible reason for the changes of vegetation growth in the Qilian Mountains. And decreasing trend in transitional region may be driven by the changes in precipitation. Increases of irrigation contribute to the upward trend of NDVI for cropland in the Hexi Corridor, reflecting that agricultural development becomes more intensive. Our study demonstrates the complexity of the response of vegetation growth in the HRB to climate change and anthropogenic activities and correspondingly adopting mechanistic ecological models capable of describing both factors is favorable for reasonable predictions of future vegetation growth. It is also indicated that improving irrigation water use efficiency is one practical strategy to balance water demand between human and natural ecosystems in the HRB.

## Introduction

Drylands are a major type of ecosystem that cover more than 40% of Earth’s land surface [1]. Severe land degradation of drylands affects directly some 250 million people in the developing world [2]. Vegetation plays an important role in maintaining dryland ecosystem functions and service [3]. It constitutes one part of a complex system, in which the atmosphere, living organisms, and soil interact closely on different scales [4]. Vegetation in drylands is highly sensitive to daily, seasonal and decadal perturbations on water availability [5], the primary sources of which are generally related to climatic change and anthropogenic activities. To better understand the roles of these two factors on dryland vegetation, researches investigating changes in vegetation dynamics are valuable [6, 7].

Monitoring vegetation by remote sensing is regarded as an effective way for resource assessment [8]. Since Landsat 1 was launched by NASA in 1972, many more satellites have been deployed to observe Earth’s resources, providing enormous quantities of information with wide spatial coverage from the intervening decades. The value of evaluating spatial ecosystem patterns and temporal processes using long-term data has been widely recognized by scientists [9]. For vegetation monitoring, the Normalized Difference Vegetation Index (NDVI), which is the ratio of the difference between near-infrared and red reflectance and the sum of these two variables, is considered as a proxy for terrestrial vegetation growth [10]. This is because there is a causal relationship between NDVI and photosynthetically active radiation absorbed by photosynthesizing tissues [11]. Trend analysis of NDVI datasets has been widely applied for detecting changes in vegetation growth on continental and regional scales (e.g., Hashimoto *et al*. [12]; Piao *et al*. [13]; Los *et al*. [14]). For dryland basins, the pattern and function of the ecosystem are highly controlled by water availability, which is influenced by the distribution and pattern of precipitation, and intensity of human use that alters natural water cycle significantly [5]. Analyzing vegetation growth trends using NDVI datasets in such basins is invaluable for improving the understanding of the relationship between water and vegetation and consequently, for implementing reasonable water resource management policies balancing the water demands of human and natural ecosystem.

The Heihe River Basin (HRB) is the second largest inland river basin in China. It is located on the northern edge of the Qinghai—Tibetan Plateau, where vegetation is vulnerable to climate change [15]. The basin is characterized by high diversity in geomorphology and irrigated agriculture in the middle reaches. The dry climate and excessive use of both river water and pumped groundwater for irrigation has led to a degradation of the ecosystem in the basin [16]. As an arid basin, water availability is one major control factor of vegetation growth. In this study of the HRB, for the purposes of exploring where and when the change of vegetation growth happened and which hydrological processes potentially drove such changes, the monotonic trend and slope magnitude of the Global Inventory Modeling and Mapping Studies (GIMMS) biweekly NDVI dataset (1982–2006) was analyzed using the Mann—Kendall test together with the Sen’s slope estimator. First, the trend of the growing season NDVI is analyzed, followed by estimations of the trend in spring, summer and autumn. The contributions of each season to the monotonic trend of growing season are discussed and conclusions drawn. Analyzing the trends of NDVI quantitatively might provide valuable insights into the response of vegetation growth in the HRB to climate change and anthropogenic activities. In our study, for the purpose of exploring the possible driving factors of changes in NDVI, besides examining its relationship with climatic variables, we also try to analyze the relation between agricultural water use and ecosystems in the HRB. From the aspect of water resources, it could lead to a better understanding of what role the anthropogenic activity plays in the changes of vegetation growth in the HRB.

## Materials and Methods

### Study area

**Fig 1:**
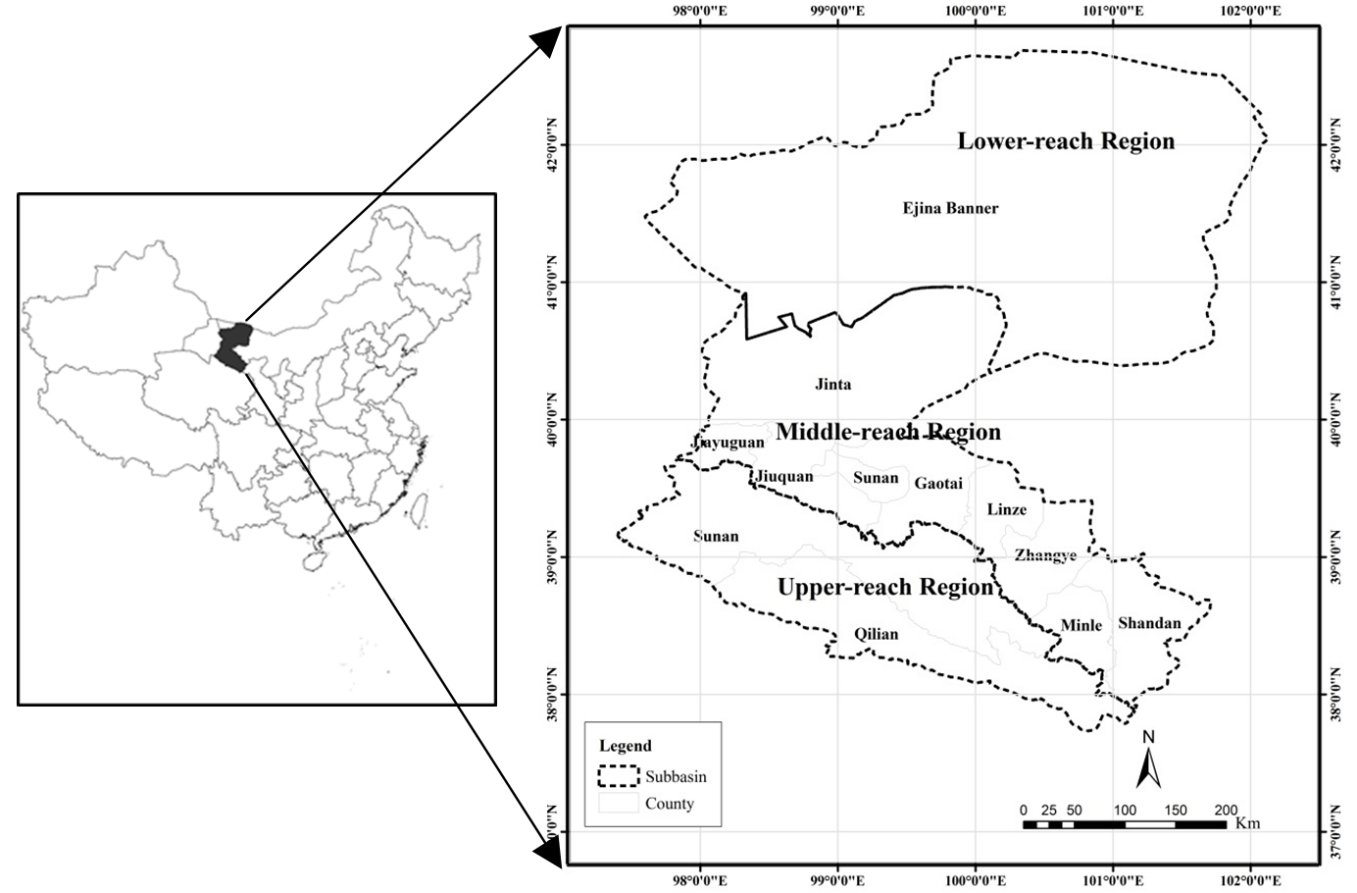
Heihe River Basin and its location in China.

**Fig 2:**
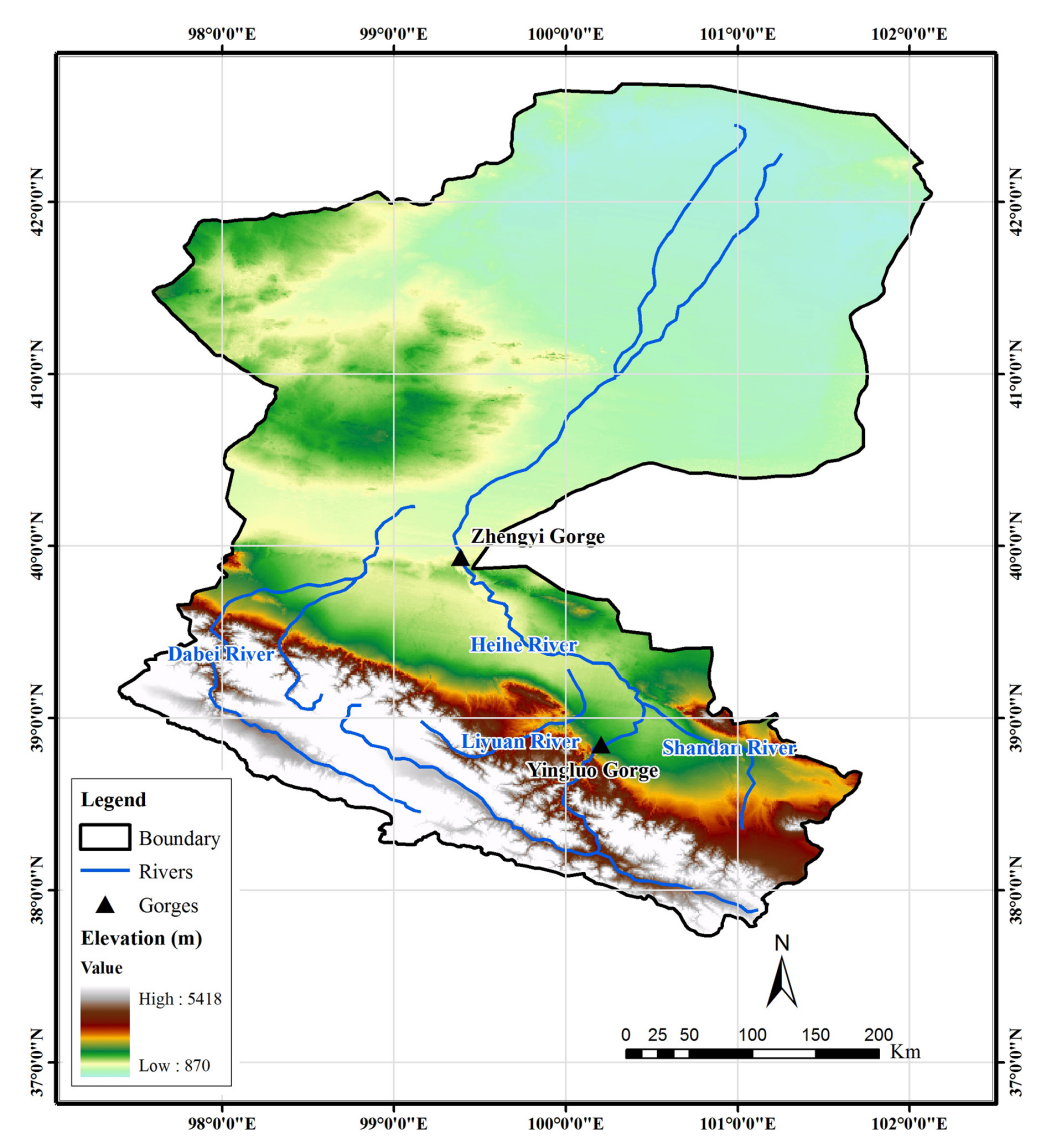
Topography and river system of the Heihe River Basin.

**Fig 3:**
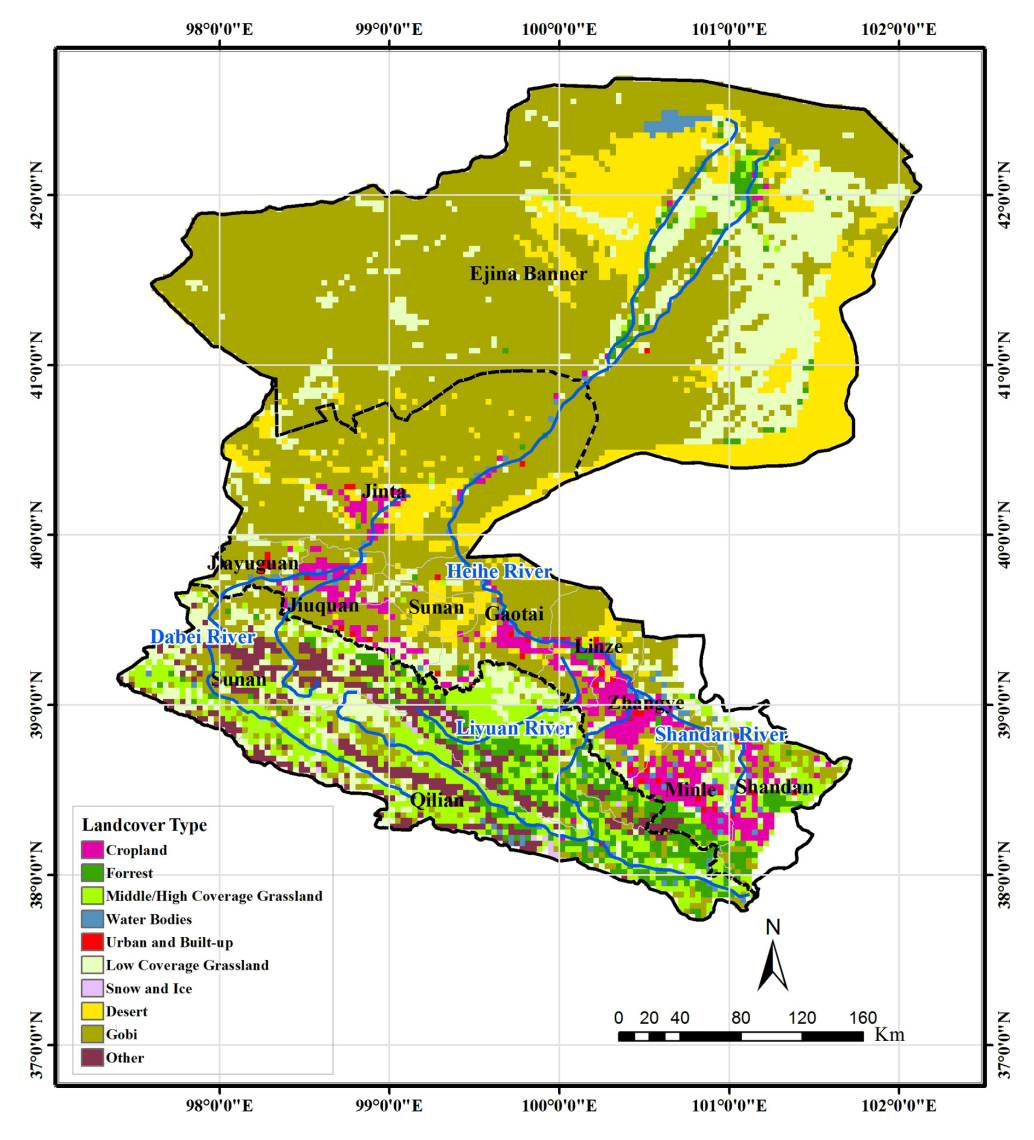
Land cover in the Heihe River Basin.

The HRB is the second largest inland basin in China, located in the arid northwest of the country (Fig 1), covering an area of about 128,900 km^2^. From the southern mountainous area to the northern high-plains area, the elevation decreases from about 5000 to 1000 m (Fig 2). The mean annual precipitation from upper-reaches region to the lower-reaches region varies from 414 to 35 mm for the period of 1960 to 2010 [17]. The land cover types of the HRB are shown in Fig 3. Based on differences of geomorphology, the basin can be divided into three regions. The upper-reaches region (including Qilian and Sunan counties) belongs to the Qilian Mountain area, which is covered mainly by natural ecosystem. The major land cover types are grassland and Gobi, as tabulated in Table 1. The middle-reaches region (including the cities of Zhangye, Jiuquan, and Jiayuguan and Minle, Shandan, Linze, Gaotai, and Jinta counties) is part of the Hexi Corridor, which is an important region for crop production in China. Gobi and cropland are the dominant land cover types. There are many man-made oases, dominated by farmland vegetation created by irrigated agriculture. Natural oases also exist in this region, scattered throughout the Gobi and desert area. Anthropogenic activities in the middle reaches are intensive, changing the land cover and consuming water resources for irrigation and domestic use. The lower-reaches region (Ejina Banner), which is extremely arid, is located on the Alxa Plateau and comprises Gobi and desert. Vegetation covers only a very small limited area and is mainly natural meadow, which has experienced severe deterioration due to the reduction of water resources caused by extensive water consumption in the middle-reaches region. As shown in Fig 2, there are two major river systems in the HRB. The mainstream of Heihe River belongs to the east system and Yingluo Gorge and Zhengyi Gorge divide the mainstream into upper, middle, and lower subbasins. The Beida River is the largest river in the west river system, which joins the mainstream of the Heihe River in Jinta County.

**Table 1:** Percentage of different land cover types in the Heihe River Basin*.

	Cropland	Forest	High/Moderate Coverage Grassland	Water	Urban
Upper-reaches Region	0.2%	13.8%	32.8%	1.1%	0.0%
Middle-reaches Region	12.7%	2.5%	4.2%	1.8%	1.1%
Lower-reaches Region	0.1%	1.5%	0.6%	0.6%	0.0%
	Desert	Snow/ Ice	Low Coverage Grassland	Gobi	Other
Upper-reaches Region	0.1%	0.7%	19.3%	14.9%	17.2%
Middle-reaches Region	11.0%	0.0%	10.8%	55.5%	0.3%
Lower-reaches Region	17.0%	0.0%	17.1%	63.2%	0.0%

*High, moderate and low coverage grassland is defined as region that percentage coverage of grassland is higher than 50%, 20% to 50% and 5% to 20%, respectively.

For the middle and lower subbasins, runoff generation is negligible [18] and water resource availability is highly dependent on the inflow at Yingluo Gorge and Zhengyi Gorge, respectively. Because of the considerable consumption of water in middle-reaches region, runoff entering the lower reaches has been reduced continuously, which has caused a series of ecological and environmental problems [19]. To ensure environmental flow in the lower reach, the National Water Diversion Project was implemented in July 2000. This restricts water use in the middle reaches and maintains the amount of river water released to the lower reaches.

### Dataset and preprocessing

The NDVI dataset used in this study was the GIMMS NDVI dataset from 1982–2006 with resolution of 8 km [20] (provided by Cold and Arid Regions Science Data Center at Lanzhou, China (http://westdc.westgis.ac.cn)). The monthly NDVI data at 8-km resolution were generated by applying a Maximum Value Composite to the two images of each month for the purposes of reducing the noise in NDVI data. Following the definition of Piao *et al*. [13], averages of NDVI data from April to October were defined as the growing season NDVI of each year. Correspondingly, spring NDVI was defined as the average monthly composite NDVI of April and May; the average monthly composite NDVI of June, July, and August was defined as the summer NDVI; and the average monthly composite of September and October was defined as the autumn NDVI. Only those pixels with average values of growing season NDVI > 0.1 were defined as area of vegetation.

### Methodology for trend analysis

For the selection of trend analysis method, De Beurs and Henebry [21] highlighted that NDVI time series are dependent on processes that are temporally correlated, causing the failure of traditionally used methods, such as ordinary least squares regression, due to the violation of underlying assumptions. The Mann—Kendall test [22, 23] is one of the most widely used non-parametric tests to detect significant trends in NDVI time series (e.g., Pouliot *et al*.[24]; de Li *et al*. [25]; Wu *et al*.[26]). It is especially effective for datasets with small sample sizes [27] and it is less sensitive to outliners [28]. As demonstrated by Piao *et al*.[13], there might be more than one trend for the NDVI time series at certain locations. In this study, the Mann—Kendall test was employed to analyze the monotonic trend of vegetation growth for the period 1981–2006, for the purposes of revealing the cumulative effects of climate change and human activity over long temporal scales. For time series data *X = {x*_*1*_, *x*_*2*_, *…x*_*n*_ *}*, the test statistic *S* is computed as

$$S=\sum_{i=1}^{\text{n-1}}\sum_{j=i+1}^{n}\text{sgn}\left(x_{j}-x_{i}\right)$$ (1)

where

$$sgn\left(x_{j}-x_{i}\right)=\left\{ \begin{array}{l} 1,x_{i}<x_{j}\\ 0,x_{i}=x_{j}\\ -1,x_{i}>x_{j} \end{array}\right.$$ (2)

Assuming that the data are independent and identically distributed, the mean and variance of the *S* statistic are given by [23]

E(S)=0 (3)

VAR(S)=n(n-1)(2n−15)/18 (4)

Considering the existence of tied ranks (equal observations) in the data, the variance of *S* is updated as

$$VAR\left(S\right)=\left[n\left(n-1\right)\left(2n-15\right)-\sum_{i=1}^{m}t_{i}\left(t_{i}-1\right)\left(2t_{i}+5\right)\right]/18$$ (5)

where *m* is the number of tied groups, and *t*_*i*_ is the number of data in the *i*th tied group. For the sample sizes > 10, the standardized normal statistic Ζ is estimated as

$$Z=\left\{ \begin{array}{l} \frac{S-1}{\sqrt{VAR\left(S\right)}},S>0\\ 0,S=0\\ \frac{S+1}{\sqrt{VAR\left(S\right)}},S<0 \end{array}\right.$$ (6)

Comparison between *Z* and the standard normal variate at the desired significance level α, allows significance of the trend to be tested. In this study, a significance level of 5% was selected, which means that if *|Z|* > 1.96, the null hypothesis of no trend would be rejected.

For those pixels for which the trend in NDVI data is significant (p < 0.05), the magnitude of changes per year can be computed using Sen’s slope estimator [29]. If a linear trend exists in the time series data, then the true slope (change per unit time) can be calculated as

$$\theta =M\text{e}dian\left(\frac{x_{j}-x_{i}}{j-i}\right),i<j$$ (7)

Positive and negative values of *θ* indicate an increasing or decreasing trend, respectively, and the absolute value of *θ* indicates the magnitude of the trend.

## Results and Discussion

### Monotonic trend in growing season

**Fig 4:**
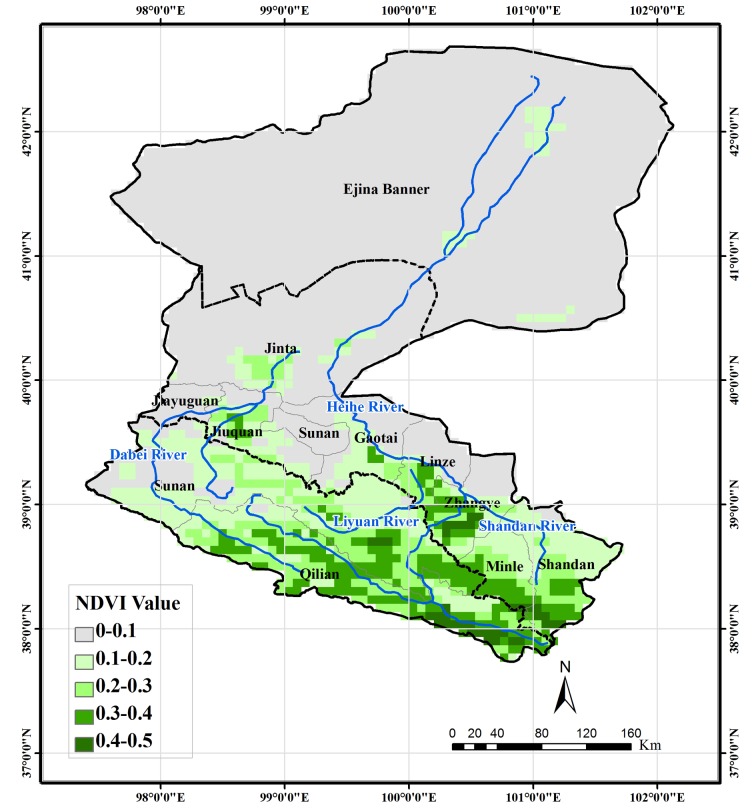
Average growing season NDVI for 1982–2006.

**Fig 5:**
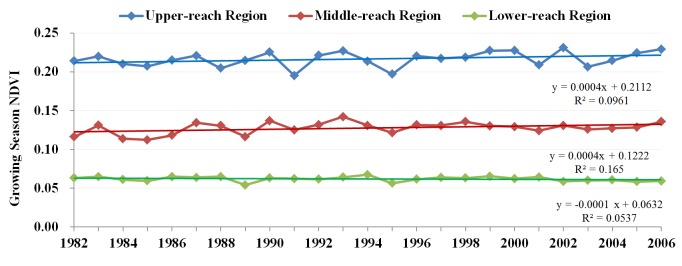
Time series of growing season NDVI in Heihe River Basin.

Fig 4 demonstrates the spatial distribution of average growing season NDVI for the period 1982–2006. Pixels with average growing season NDVI > 0.1 are defined as regions covered by vegetation. The vegetation area is located mainly in the upper- and middle-reaches regions. In the upper-reaches region, forest and high/middle cover grass are the dominant vegetation types, as shown in Fig 3. For the middle-reaches region, the area with average growing season NDVI > 0.1 is covered mainly by cropland and natural grassland near cropland and rivers. In the lower-reaches area, only a limited area around the terminal lake is covered by vegetation. Fig 5 shows the time series of growing season NDVI for the period 1982–2006. Generally, in the upper- and middle-reaches region, a weak increasing trend can be seen, whereas in the lower reach region, a weak decreasing trend can be observed.

**Fig 6:**
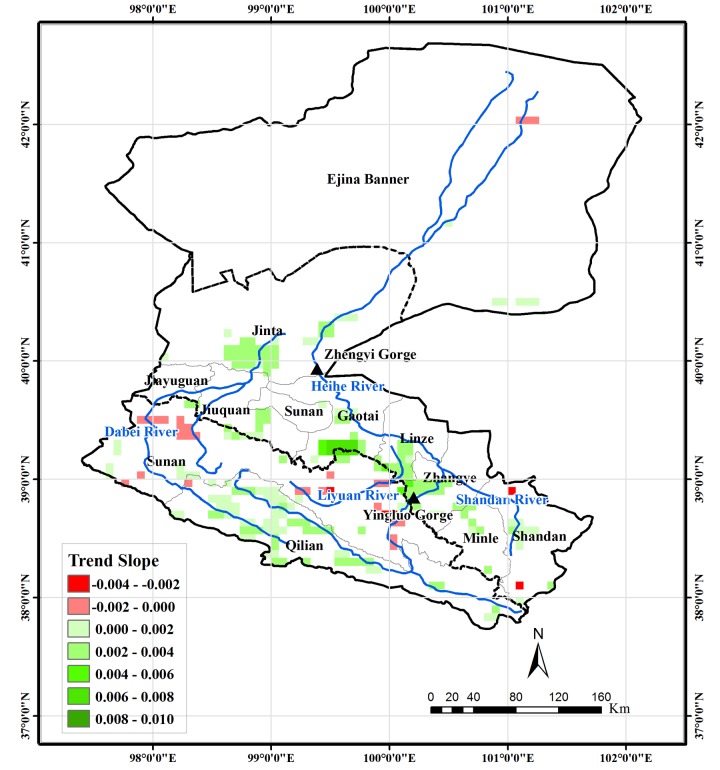
Vegetation areas for which the trend of growing season NDVI for 1982–2006 is significant at the 0.05 level and their trend slope.

The Mann-Kendall test was used to analyze the trend of growing season NDVI of pixels covered by vegetation. The results indicate that 10.1% and 1.6% of the area in the HRB is found to exhibit an increasing and decreasing trend, respectively, at the 0.05 significance level (Fig 6), and the maximum magnitude of increase and decline is 0.066/10a and 0.026/10a, respectively. The headwater areas of both the Heihe and Beida Rivers in Qilian County show a low-to-medium (lower than 0.033/10a) increasing trend. A medium-to-high (higher than 0.033/10a) increasing trend is observed in the some parts of the oases near both the east and west river systems in the middle-reaches region. Those pixels showing a decreasing trend are located mainly in the transitional region between the Qilian Mountains (the region between the south border of the basin and the north edge of the Qilian Mountain) and the Hexi Corridor (the flat region around river system in the middle-reaches region), including those in the northwestern part of Sunan County in the west river system, Liyuan River and near the Yingluo Gorge. Limited area in the lower-reaches region near the terminal lake shows low-to-middle decrease trend. The results of the trend analysis are generally similar to those found by Ma and Veroustraete [30], who used linear regression on the same NDVI dataset from 1982–2001 to analyze the vegetation changes in the HRB. This implies that there is no significant difference in NDVI trend for the periods 1982–2001 and 2002–2006.

### Trend in spring, summer, and autumn

**Fig 7:**
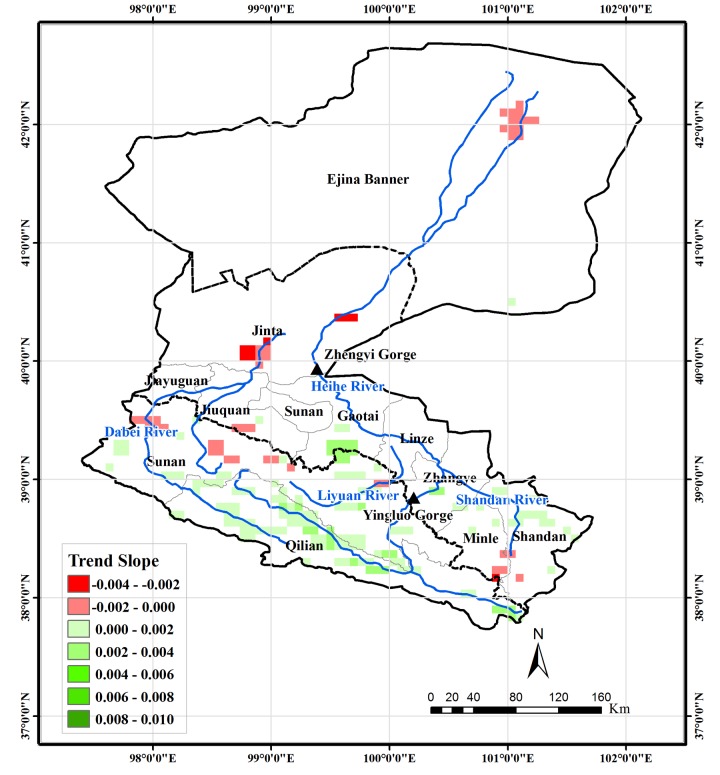
Vegetation areas for which the trend of NDVI in spring for 1982–2006 is significant at the 0.05 level and their trend slope.

**Fig 8:**
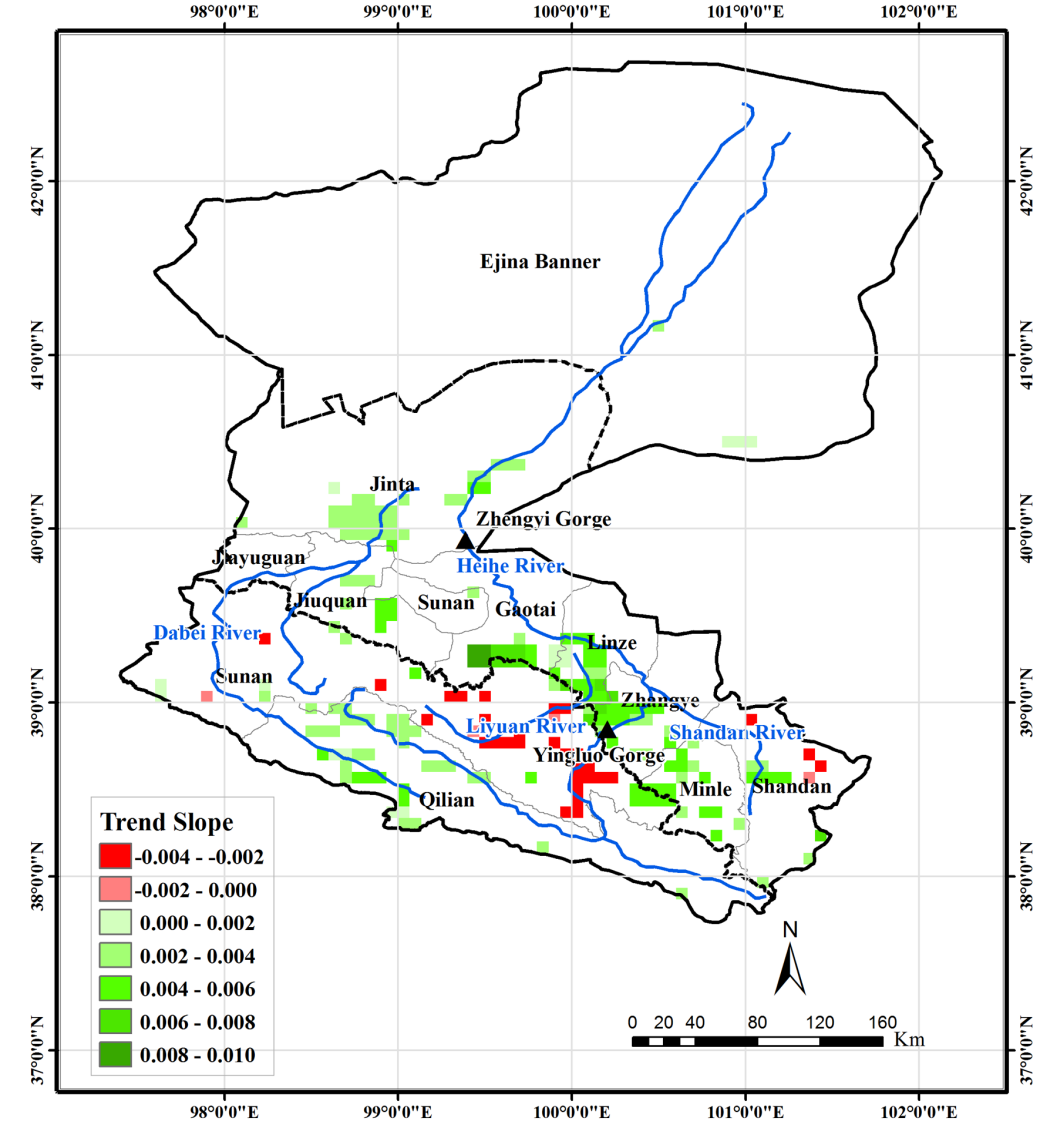
Vegetation areas for which the trend of NDVI in summer for 1982–2006 is significant at the 0.05 level and their trend slope.

**Fig 9:**
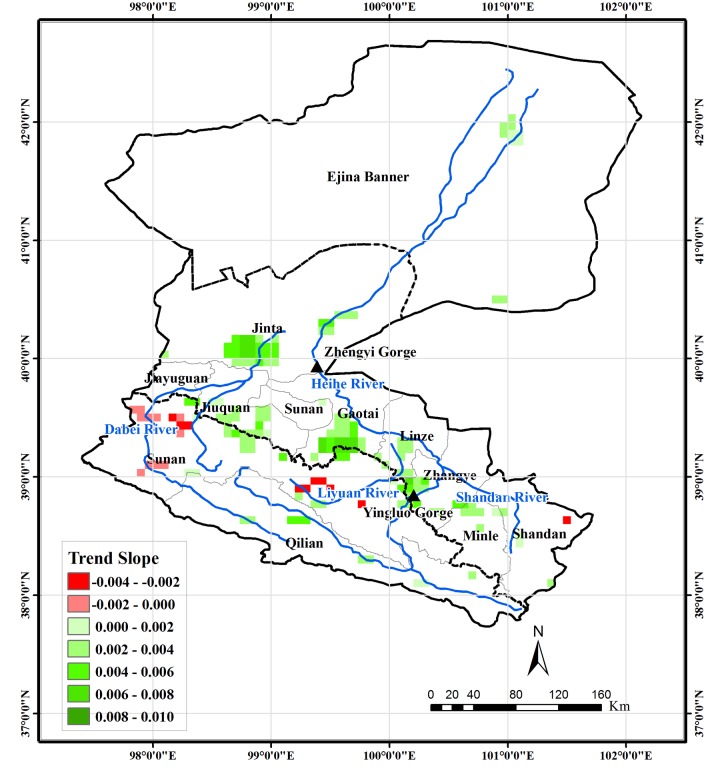
Vegetation areas for which the trend of NDVI in autumn for 1982–2006 is significant at the 0.05 level and their trend slope.

To explore the contributions of spring, summer, and autumn to the monotonic trend in growing season, which was not discussed in the study of Ma and Veroustraete [30], the trends of NDVI in the three seasons were analyzed in this study and the results were presented in Figs 7–9. In spring, the headwater area in Qilian County also shows an upward trend. For the oases in the middle-reaches region, only the pixels in south Gaotai County and a very few pixels close to the river are detected as exhibiting an increasing trend. The areas showing a decreasing trend are mainly located in southern Minle and Shandan Counties, northern areas of Sunan County near Jiayuguan and Jiuquan, the oasis in Jinta County, and the vegetated area around the terminal lake. In summer, the spatial distribution of NDVI trend is very similar to that of the monotonic trend in the growing season. However, the magnitude of changes is larger than those in the entire growing season. In the Liyuan River and upstream area of the HRB near the Yingluo Gorge, both the spatial extent and magnitude of the decreasing trend is larger than for the entire growing season. In autumn, the oases in the middle-reaches region show a similar increasing trend to that of the entire growing season. The areas with a downward trend are located mainly in the Liyuan River area and northwestern parts of Sunan County.

### Possible factors driving changes in vegetation growth

Generally, the spatial pattern of trend of growing season NDVI is that in the upstream area of Qilian Mountain and Hexi Corridor exhibit an increasing trend, whereas, in the transitional region between Qilian Mountains and Hexi Corridor, where vegetation is natural, exhibits a decreasing trend. Natural vegetation in a dryland ecosystem is very sensitive to precipitation. Three meteorological stations were selected to represent the characteristics of temporal variation of precipitation in the Qilian Mountains, the transitional region, and Hexi Corridor. The average annual precipitation for 1960–2006, 1980–1989, 1990–1999, 2000–2006 are tabulated in Table 2 (Data obtained from [31]). The precipitation in the Qilian Mountains shows a generally increasing trend on the decadal scale for 1980 to 2006, and the 10-year average values are higher than that for 1960 to 2006. This could be one possible reason for the increasing trend in vegetation growth. The increasing trend of NDVI can be attributed mainly to the increments in spring and summer. In the upper- and middle-reaches of the HRB, increasing trends in temperature has been observed [32], especially in the Qilian Mountains. This is potentially another factor driving the increasing trend in NDVI. As indicated by Piao *et al*. [33], the warming trend of temperature triggers an earlier vegetation green-up in the Northern Hemisphere. In the transitional region, the value of average annual precipitation in 1990–1999 and 2000–2006 are lower than for 1960 to 2006. Niu and An [34] also detected a decreasing trend on an annual scale. As this region is remote and difficult to access, compared with the Hexi Corridor where agricultural development is intensive, the human activities are minor. Therefore, the impact of anthropogenic activities on vegetation greenness is not significant. Precipitation is the main source of water for vegetation growth and consequently, the decline in precipitation is considered as one of the principal reasons for the deceasing trend in NDVI in some parts of this region.

**Table 2:** Average annual precipitation for different periods at three meteorological stations (unit: mm).

	Qilian Mountain	Transitional Region	Hexi Corridor
Station	Zhamashike	Wafangcheng	Yingluo Gorge
1960–2006	453.3	443.2	182.7
1980–1989	465.1	470.3	174.1
1990–1999	464.7	412.9	202.5
2000–2006	472.1	435.2	177.6

**Fig 10:**
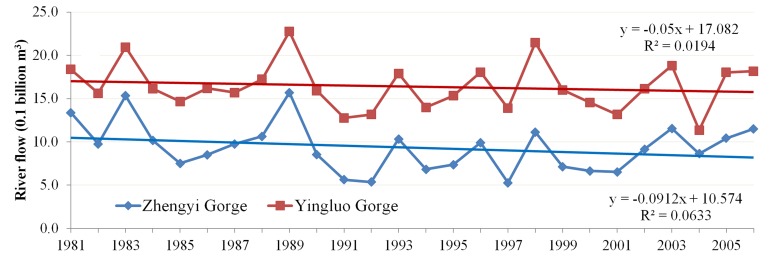
Annual river flows at the Yingluo Gorge and Zhengyi Gorge for 1981–2006 and their trend lines.

For the middle-reaches area located within the Hexi Corridor, analysis by Li and Xu [32] indicated an absence of any significant trend in precipitation. The data in Table 2 also reveal the lack of any monotonic trend of increase or decrease on the decadal scale. Oases in this region consist mainly of cropland and surrounding natural vegetation that rely on irrigation for growth. The irrigation water comes from river water in the mainstream of Heihe River and pumped groundwater. As runoff generation in this region is minor, differences of river flow between Yingluo Gorge and Zhengyi Gorge can be treated as an indicator of how much water are extracted for irrigation purposes. The annual volume of river flow at Yingluo Gorge and Zhengyi Gorge for 1981–2006 and their trend lines are depicted in Fig 10. The slopes of both trend lines are negative and the absolute value for the slope of Zhengyi Gorge is larger than Yingluo Gorge. All these facts indicate that the difference of river flow between the Yingluo Gorge and Zhengyi Gorge is increasing; i.e., in other words, the amount of water extracted for irrigation in the Hexi Corridor has risen from1981 to 2006 generally. In this region, it is also found that the increases of NDVI in summer and autumn, which are the growing and maturing periods for crops, make the greatest contributions to the general upward trend of NDVI in the growing season. Therefore, the upward trends in summer and autumn indicate that agricultural development intensity increases, which is supported by the increase in water extracted for irrigation. Furthermore, it is indicated that influences of human activity are stronger than climate changes on vegetation growth in the HRB. For reasonable predictions of future vegetation growth in arid basins, adopting mechanistic ecological models capable of describing both climate changes and anthropogenic activities, especially the water resource reallocation due to human consumption, is favorable, as in arid basins, water availability is one major controlling factor on the health of ecosystem [5].

Pumping groundwater and direct use of river water for crop production changed status of hydrological processes in the middle- and lower-reaches regions. The spatial distribution of groundwater level has been greatly influenced by pumping in the middle-reaches region (Ji *et al*., 2006 [35]). For the cropland and surrounding natural oasis, the irrigation water goes back to the ground and increase soil moisture. In natural oases far from cropland in the Hexi Corridor, following the decrease of ground water level, the soil moisture also reduces. It can be considered as one possible reason of downward trend of vegetation growth near the cross point between the Liyuan River and the border between upper and middle-reaches region, which belongs to the south edge of the Hexi Corridor. The source of groundwater and terminal lake in lower-reaches region is river water from Zhengyi Gorge. Lower releases of streamflow from middle-reaches region cause decreases of groundwater level in the lower-reaches region. Zhu *et al*. (2012) [36] showed that the vegetation growth highly rely on groundwater. Therefore, the decrease of NDVI near the terminal lake is also a possible outcome of excessive use of water for irrigation in the middle-reaches region. Undoubtedly, the agricultural water use has negative influences on natural vegetation in both middle- and lower-reaches region. However, as an important area for crop production in China, providing sufficient water for irrigation is necessary to ensure national food security. Apparently, there is a competition of water recourses between irrigated agriculture and ecosystem. To explore the possibility of reducing the amount of irrigation water for maintaining the health of natural ecosystem, the agricultural water use efficiency in the middle-reaches region is evaluated through the index of the effective utilization coefficient of irrigated water, which is the ratio of water being effectively used for crop growth and being irrigated. The coefficients for three cities (obtained from [37]) are tabulated in Table 3. Compared with the value of the Haihe Basin (obtained from [38]), which is also located in north China and faces the problem of severe water scarcity, the irrigation efficiency is low in HRB. In this context, in the middle-reaches region, reducing pumping groundwater and increasing river water release downstream by improving irrigation water use efficiency is desirable to balance water demand between human and natural ecosystems, which provide a possible approach for improving sustainable development in HRB.

**Table 3:** Comparison of effective utilization coefficient of irrigated water.

Zhangye City	Jiuquan City	Jiayuguan City	Haihe Basin
0.49	0.53	0.52	0.64

## Conclusions

In this study, changes in the vegetation growth of the HRB, the second largest inland basin in China, were detected using the GIMMS biweekly NDVI dataset. An increasing trend was observed in some parts of the upstream area of the Qilian Mountains and the Hexi Corridor in the middle-reaches region. For the transitional region between the Qilian Mountains and the Hexi Corridor, where the oases are mainly natural, a decreasing trend was detected in some parts. To explore the possible causes of the changes in vegetation growth, the trends of spring, summer, and autumn were analyzed. The increase of precipitation and temperature in the area of the Qilian Mountains are possible reasons for upward trend in NDVI. The decrease in precipitation in the transitional region could potentially drive the downward trend of NDVI. The increase of water extraction for irrigation in the Hexi Corridor contributes to the increasing trend of NDVI in the cropland and decreasing trend in remote natural oases located in middle-reaches region and vegetation near the terminal lake of the HRB. The differences in trend of growing season NDVI among different regions demonstrate the complexity of the responses of vegetation growth in the HRB to climate change and anthropogenic activities. From the viewpoint of water resources management, reducing pumping groundwater and use of river water for irrigation via improving water use efficiency is one practical approach to enhance the health of natural ecosystems in middle- and lower-reaches regions of the HRB. To obtain a greater understanding of the relationship between biotic processes and elements of the water cycle within this basin, an analysis of observations covering the entire basin with high spatial resolution, such as could be obtained by the Heihe Watershed Allied Telemetry Experimental Research (HiWATER) (http://hiwater.westgis.ac.cn/english/) project, are extremely desirable.
